# Climate change, climatic variation and extreme biological responses

**DOI:** 10.1098/rstb.2016.0144

**Published:** 2017-05-08

**Authors:** Georgina Palmer, Philip J. Platts, Tom Brereton, Jason W. Chapman, Calvin Dytham, Richard Fox, James W. Pearce-Higgins, David B. Roy, Jane K. Hill, Chris D. Thomas

**Affiliations:** 1 Department of Biology, University of York, Wentworth Way, York YO10 5DD, UK; 2 Butterfly Conservation, Manor Yard, East Lulworth, Wareham BH20 5QP, UK; 3 AgroEcology Department, Rothamsted Research, Harpenden AL5 2JQ, UK; 4 Centre for Ecology and Conservation, and Environment and Sustainability Institute, University of Exeter, Penryn TR10 9EZ, UK; 5 British Trust for Ornithology, The Nunnery, Thetford IP24 2PU, UK; 6 Conservation Science Group, Department of Zoology, University of Cambridge, Downing Street, Cambridge CB2 3EJ, UK; 7 Centre for Ecology and Hydrology, Wallingford OX10 8BB, UK

**Keywords:** Aves, butterfly, climatic risk, Lepidoptera, moth, weather

## Abstract

Extreme climatic events could be major drivers of biodiversity change, but it is unclear whether extreme biological changes are (i) individualistic (species- or group-specific), (ii) commonly associated with unusual climatic events and/or (iii) important determinants of long-term population trends. Using population time series for 238 widespread species (207 Lepidoptera and 31 birds) in England since 1968, we found that population ‘crashes’ (outliers in terms of species' year-to-year population changes) were 46% more frequent than population ‘explosions’. (i) Every year, at least three species experienced extreme changes in population size, and in 41 of the 44 years considered, some species experienced population crashes while others simultaneously experienced population explosions. This suggests that, even within the same broad taxonomic groups, species are exhibiting individualistic dynamics, most probably driven by their responses to different, short-term events associated with climatic variability. (ii) Six out of 44 years showed a significant excess of species experiencing extreme population changes (5 years for Lepidoptera, 1 for birds). These ‘consensus years’ were associated with climatically extreme years, consistent with a link between extreme population responses and climatic variability, although not all climatically extreme years generated excess numbers of extreme population responses. (iii) Links between extreme population changes and long-term population trends were absent in Lepidoptera and modest (but significant) in birds. We conclude that extreme biological responses are individualistic, in the sense that the extreme population changes of most species are taking place in different years, and that long-term trends of widespread species have not, to date, been dominated by these extreme changes.

This article is part of the themed issue ‘Behavioural, ecological and evolutionary responses to extreme climatic events’.

## 1. Introduction

Climate is an important determinant of species range, population change, abundance, phenology and biotic interactions [1–4]. The precise sequence of climatic events and the time of year when these events occur may affect whether a species' biological response is rapid life cycle development and increased reproduction leading to population growth, or increased mortality leading potentially to extinction. In the context of this paper, climate change represents a change to the frequency, severity and sequences of different weather events, which may lead to increases in the frequency of some forms of extreme events such as those associated with heat, drought or flooding, but decreases in others, such as those associated with cold [5]. It has been suggested that such extreme events may generate substantial population responses and community transitions, and that these rare events could be as important in determining ecological responses to climate change as are long-term changes to the average climatic conditions that a population experiences [6]. However, rigorous assessment of the frequencies and impacts of extreme population responses are constrained by the limited availability and spatial/taxonomic coverage of long-term population data [7], and also because a given sequence of climatic events will not necessarily generate a consensus response in organisms [6] due to interspecific differences in species' ecological traits and sensitivity to climate. Previous studies have highlighted the individualistic nature of species' responses to different aspects of the climate at different times of year [8–11] although, in general, such studies have focused on describing responses to climatic means, rather than extremes. Here, we assess the extent to which extreme population responses are individualistic (i.e. whether there is an agreement among species about which years are ‘extreme’), and evaluate whether extreme population responses are important determinants of species' long-term population trends.

Extreme climatic events (ECEs), by their very nature, are outside of the norm experienced by organisms and to which species may be (locally) adapted. As such, we hypothesize that extreme events are more likely to drive negative rather than positive population changes. Therefore, we also assess whether extreme species' responses are more frequently negative, and whether these events are commonly associated with unusual climatic conditions. Previous approaches to understanding the importance of ECEs for biological communities have been either to identify such an event (e.g. a drought) and then see if some or many species responded to it or, alternatively, to seek an explanation for one-off extreme population changes that have been observed [12]. Such studies have provided strong evidence of population crashes in response to unusual climatic conditions, especially in relation to extreme droughts, winter freezing, unseasonal cold and excessive heat ([5,13–19], cf. coral reef bleaching and anoxia in aquatic systems [20,21]). However, there is potential that the results could be unrepresentative if the choice of year, climatic event or species under consideration have been influenced by the events themselves. Hence, the choice of study species may not be appropriate to elucidate the frequencies of rare events or their long-term importance during a period of climatic change. As Bailey & van de Pol [6] and van de Pol *et al*. [22] discuss, a major drawback of many studies linking ecological and climatic extremes has been a focus on the impacts of single climatic events, over short time periods, leaving questions remaining about the long-term implications of extreme events (but see [23]). Here, we remove these potential biases by taking a multi-species approach, analysing data over a relatively long, continuous time period to find out whether extreme population changes tend to take place in, or following, years that are also climatically extreme. To do this, we utilize long-running population dynamic data at a national scale for 238 species from two broad taxonomic groups (31 birds and 207 Lepidoptera in England), to identify group- and species-specific differences in population responses to ECEs. For each species, we identify years when they show unusually high levels of population growth or decline, and assess whether the proportion of species exhibiting extreme population changes each year are associated with particular climatic conditions.

Population growth rates of species with similar life histories (e.g. clutch sizes or survival rates) have the potential to be highly synchronized [24,25], while differences in life history can desynchronize dynamics across species [24,25]. Thus, we contrast the timing of extreme responses of birds and Lepidoptera, with the expectation that we will observe similar temporal responses within, but not between, these two taxonomic groups. We then go on to identify consensus years where an unusually large proportion of species experiences extreme population changes, and assess whether these consensus years tend to coincide with extreme climate conditions in the same and/or previous year. Although the importance of ECEs to population dynamics is widely discussed in the ecological and climate change literatures [6], the extent to which these events do or do not predict long-term population trends has not been assessed robustly. There is no necessary link between the two, although there is certainly the potential for ECEs to cause long-term population changes (e.g. [26]). There may be no link because extreme events, by definition, are rare, and an extreme change in one year may have very little impact on the average rate of population growth or decline over a longer period. Alternatively, it is possible that the cessation of some kinds of ECEs (which previously either constrained populations, or generated periodic increases in reproduction) may be as important to long-term population changes as an increased frequency of previously rare or wholly novel conditions. The influence of such events may only be seen in population time series of long duration. Therefore, we consider empirically whether the long-term population trends of species (over four decades) are linked to the extreme population responses that they exhibit over the entire period.

For linguistic simplicity, throughout this article we refer colloquially to population ‘crashes’ (steep year-to-year national population declines—see Material and methods), population ‘explosions’ (rapid increases), ‘bad years’ (years in which crashes take place), ‘good years’ (years in which explosions take place), ‘consensus bad years’ and ‘consensus good years’ (years with a significant excess of population crashes or explosions, respectively). We consider the hypotheses that:

(i) most years are associated with extreme population changes in some species (because biological responses to the environment differ among individual species and between higher taxonomic groups);
(ii) population crashes tend to be more frequent than population explosions during periods of rapid climatic change (as new environments are experienced), and crashes are more extreme than explosions (because the latter are constrained by the intrinsic rate of population growth whereas, in principle, all individuals could die simultaneously);
(iii) consensus years are associated with unusual climatic conditions in the same or previous year; and
(iv) long-term population trends are correlated with extreme population responses.

## 2. Material and methods

We define our study area as mainland England, chosen because a large quantity of reliable, long-running annual count data for birds and Lepidoptera (butterflies and macro-moths) are available at this spatial extent. Although Lepidoptera data are also available from the rest of the United Kingdom, we restricted our analyses to match the spatial extent of the bird data, so that the two groups could be directly compared. We conducted our analyses using *R*, v. 3.1.0 [27].

### (a) Species data

For each species we obtained (for birds) or calculated (for Lepidoptera) national indices of abundance across England. We then used these data to calculate year-to-year changes in population index and long-term abundance trends, as described below.

We obtained species data for butterflies, moths and birds from the UK Butterfly Monitoring Scheme (UKBMS; [28]), the Rothamsted Insect Survey (RIS; [29]), the Common Bird Census (CBC; [30]) and the Breeding Bird Survey (BBS; [31]). These schemes are national networks of standardized count surveys using either territory mapping (CBC), fixed-location line transects (UKBMS and BBS) or fixed-location light traps (RIS). Butterfly count data (species' abundances for individual sites each year) were collected from 1665 sites spanning the years 1976–2012. Macro-moth count data (species' abundances for individual sites each year) were from 295 sites spanning the years 1968–2012. National population indices of birds spanned the years 1968–2012, combining data from the CBC, which ended in 2000, with data from the BBS which started in 1994 (see [10]). There were no bird data for the year 2001 because foot-and-mouth disease severely restricted access in that year.

We included butterfly and moth species for which there were at least five sites with non-zero counts in every year of the time series (37 years for butterflies and 45 for macro-moths), and birds which were sufficiently well monitored by both CBC and BBS surveys. Migrant birds and true-migrant Lepidoptera were excluded, because extreme population changes of such species may not be a result of climate experienced solely in our study area, although the English populations of the most mobile species will still experience some exchanges with regions outside the study region. Thus, we included 178 macro-moth species, 29 butterfly species and 31 bird species in our analyses (listed in electronic supplementary material, table S1). Butterflies and moths were analysed together as they belong to one taxonomic order (Lepidoptera), while we hypothesize that birds will differ in their response to climate, and so they were analysed separately.

For each macro-moth and butterfly species, we obtained national indices of abundance in two steps: first, for each species, we related the species' annual count data per site to year (as a fixed factor) in a generalized mixed effects model with site as a random intercept, and a Poisson error distribution. We then took the fixed (year) coefficients from each species' model, which quantify the annual relative abundances of species.

**Figure 1.**
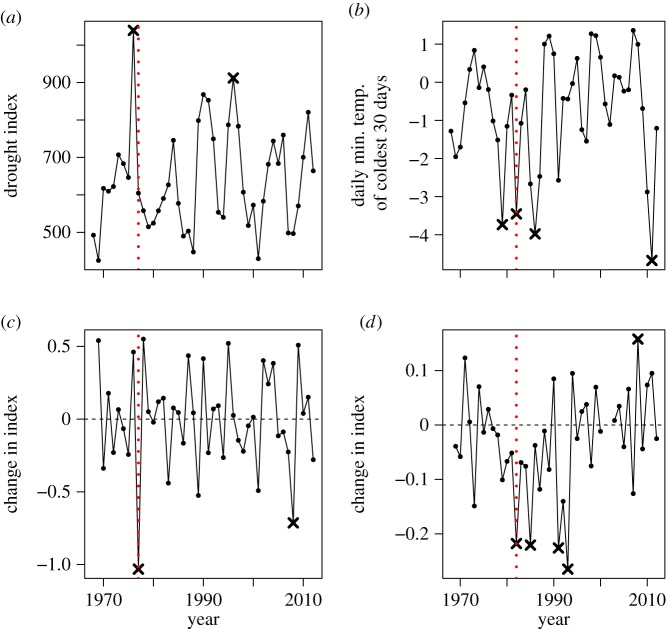
Exemplar climatic variables and species to illustrate our approach. The plots show how we identified extreme climatic events (*a*,*b*) and species responses (*c*,*d*). The vertical (red) dashed lines represent the largest consensus year, where an extreme number of Lepidoptera (*a*,*c*) and birds (*b*,*d*) experienced population crashes. (*c*,*d*) Year-to-year changes in index of two example species, chosen as they experienced the greatest crashes in the largest consensus year for each species group: the mottled grey moth *Colostygia multistrigaria* (*c*) and the tree sparrow *Passer montanus* (*d*). Values below zero in (*c*,*d*) indicate negative population growth, and values above zero indicate positive growth. In each panel, extreme years (outliers) for climate and species are represented by black crosses. (Online version in colour.)

We calculated year-to-year changes in the index by subtracting the log_10_ index value in year*_t_* from the log_10_ index value in year*_t_*_+1_ (figure 1*c*,*d*). We also calculated each species' long-term change in abundance over our study period as the slope of a linear model relating national indices of abundance against year.

### (b) Climate data

We downloaded gridded climate data for the period 1965–2011 from the UK Met Office website (www.metoffice.gov.uk/climatechange/science/monitoring/ukcp09), supplemented with data for 2012 obtained directly from the Met Office. These data provide daily estimates of minimum and maximum temperature, and monthly rainfall estimates, at a spatial resolution of 5 × 5 km on the Ordnance Survey National Grid reference system. From these data, we derived a set of 13 annual climate variables that may correlate either directly (physiological limits) or indirectly (i.e. relevance for habitat, food or host plants) with the population dynamics of our study species (electronic supplementary material, tables S1 and S2). Further analyses were conducted on spatial mean values, calculated across England, for each year in the population time series.

We reduced levels of collinearity in the climate data using the following procedure, whereby highly correlated variables (Pearson's |*r*| > 0.7) were sequentially removed. For each pair of correlated variables in turn, starting with the most strongly correlated pair, the variable that was collinear with the greatest number of other climate variables was removed; where a pair of variables was collinear with the same number of other variables, the one with the largest mean absolute correlation was removed. The seven retained climate variables included measures of rainfall seasonality, drought, temperature range, growing degree days as well as coolness and hotness (table 1).

**Table 1.** Climate variables used in the analyses. ‘Extreme’ years are listed in which the England-wide average conditions were greater than (‘positive extreme’) or less than (‘negative extreme’) twice the median absolute deviation from the median. With the exception of the drought index, each variable was calculated over the 12-month period from 1 September to 31 August (i.e. 1979 corresponds to the period 1 September 1978 to 31 August 1979). For the drought index, calculations ran over an 18-month period (beginning 1 March) in order to capture water deficit accumulated over successive hot and dry springs/summers.

variable	abbreviation	units	positive extreme	negative extreme	description
rainfall wettest month	WETTEST	mm			rainfall of the wettest calendar month
rainfall seasonality	RAINSEASON	mm	1979, 1990, 1995		rainfall contrast across seasons [32]: ∑*s* = 1..4 |Rs–RT/4|/RT, where Rs is rainfall in season s, and RT is total annual rainfall
drought index	DROUGHT	mm	1976, 1996		accumulated water deficit, where a deficit is defined by monthly Hargreaves PET > monthly rainfall. Months with excess rainfall reduce the deficit, but only up to field capacity. The drought index is the maximum water deficit recorded during spring/summer of the reference year
growing degree days	GDD5	°C	2007		annual sum of degrees by which daily mean air temperature exceeds 5°C
annual temperature range	TEMPRANGE	°C			annual maximum air temperature minus annual minimum air temperature
daily minimum temperature of coldest 30 days	COLD30	°C		1979, 1982, 1986, 2011	mean of daily minima over coldest consecutive 30-day period
daily maximum temperature of hottest 30 days	HOT30	°C	1976, 1995, 2006		mean of daily maxima over hottest consecutive 30-day period

We summarized temporal variation in these variables by plotting the first three axes of a principle components analysis, using the ‘PCA’ function of the ‘FactoMineR’ package in R [33]. For comparison with the species data, we computed the three-dimensional Euclidian distance of each year from the origin of the PCA, which is a measure of how unusual a year was in terms of the unique combinations of climate in that year.

### (c) Statistical analyses

#### (i) Defining and describing extreme events

There are many different approaches to defining an extreme event, including identifying observations at the tails of a given frequency distribution (typically, and arbitrarily, selecting 5 or 10% of the data), or those above or below an absolute critical threshold (e.g. [22,23,34–36]). In the context of our study species, the percentile approach would mean that all species would be assigned at least one good year and one bad year, irrespective of the spread of year-to-year changes in index across their study periods. We therefore identified extreme changes as those beyond species-specific thresholds, defined by the median value over the study period ± two median absolute deviations (MAD) [37], according to equation (2.1):2.1
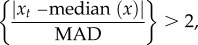
where *x_t_* is a species' year-to-year change in index in year *t*, and *x* is the full time series of the species' year-to-year changes in index. Thus, we defined explosions and crashes relative to the median in a symmetrical fashion (figure 1), because we found no consistent asymmetries in species' changes in index (robust measure of skewness [38]: mean across all species = −0.02 (range = −0.47 to 0.44)).

We used this same approach to define extreme climate years, according to the seven climate variables described in table 1.

We investigated the degree of association between the occurrences of explosions/crashes across all years by correlating the proportion of Lepidoptera (or birds) experiencing population crashes each year to the proportion of Lepidoptera (or birds) experiencing population explosions, using Spearman's rank correlations. We then identified ‘consensus’ years, during which more species experienced extremes in the same direction (crash or explosion) than would have been expected by chance, based on a one-tailed exact binomial test using the observed frequencies of crashes and explosions within each group (Lepidoptera or birds, with Bonferroni correction for multiple-year testing).

To investigate whether population trends were related to extreme population responses, each species' long-term change in abundance was plotted against the maximum absolute population crash or explosion (that qualified as an extreme) for that species, and also against the mean of all extreme crash or explosion events experienced by that species during the study period. These two metrics should reveal whether extreme population changes have a long-term effect on population size (e.g. if numbers were high and crashed in year 5, and stayed low thereafter, there would be a negative relationship between year and population size; but if there was density-dependent recovery, there would be no relationship, or even a positive relationship). Species that did not show any extreme population change values (*n* = 2 birds, 27 moths and three butterflies) were excluded from this analysis.

#### (ii) Linking population extremes to climate

Each period of population change refers to the change in index values (counts) between years, for example between 1968 and 1969. Each climatic year also corresponds to a 12-month period (with the exception of drought index), such that the climate referred to as ‘1969’ refers to the climatic period from 1 September 1968 to 31 August 1969 (table 1). The data for these two years would be compared to consider direct (lag 0) effects of climate on population change (e.g. the 1969 climate compared to the 1968–1969 population change). Population crashes and explosions were also related to climatic conditions in the previous year (climatic year ‘1968’, lag 1). We considered lagged effects because impacts of ECEs can be direct (e.g. population growth in response to a warm summer), or delayed by a year or more due to species' long generation times or through altered natural enemy or food abundances.

First, we examined whether there were associations between species' consensus years and extreme climate years (table 1) using a Fisher's Exact-Boschloo test. For this test, we used a contingency table which summed the number of occasions when species consensus years coincided (or not) with years with extreme climate (with up to 1-year lag). Then, in order to investigate more generally if extreme population responses were associated with ECEs, the summed number of Lepidoptera or bird species experiencing an extreme event (crash or explosion) each year was plotted against (i) the three-dimensional Euclidian distance from the PCA origin, (ii) drought index, and (iii) daily minimum temperature of coldest 30 days, as we hypothesized these would be the main drivers of population change for our focal species groups. In each case, we accounted for a direct and a 1-year lagged effect. As such, statistical inference was Bonferroni-corrected for multiple (*n* = 12) tests.

## 3. Results

### (a) Extreme population changes

**Figure 2.**
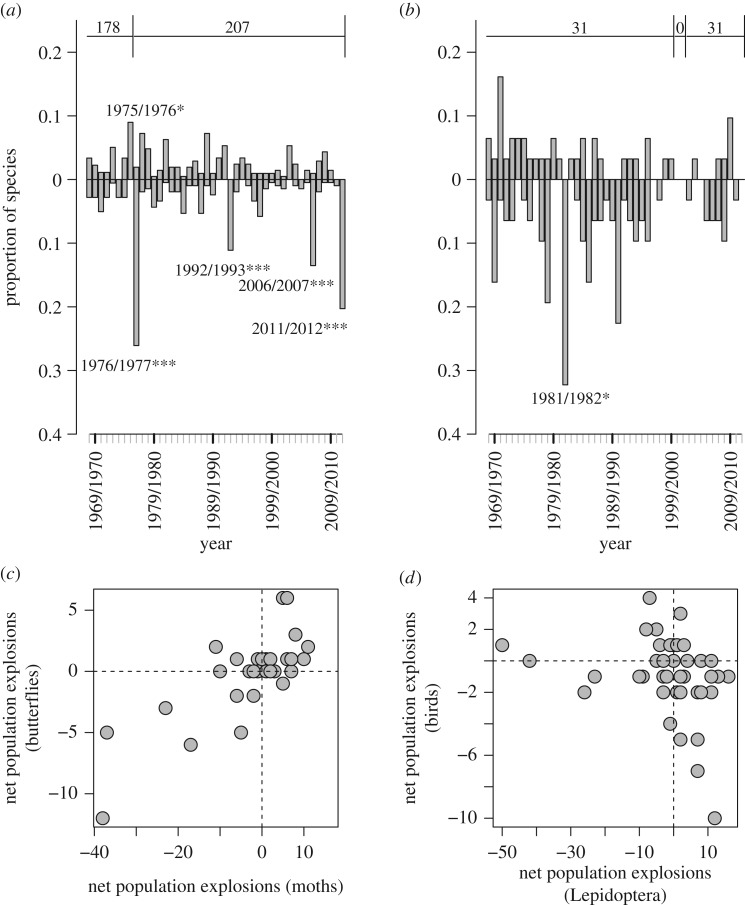
Annual extreme population changes of English Lepidoptera and birds. Upper panels: proportion of Lepidoptera ((*a*); butterflies and macro-moths) and bird species (*b*) experiencing a population explosion (upwards bars) or crash (downwards bars). Asterisks denote significance of consensus years (*p < 0.05; ***p < 0.0001; Bonferroni-corrected for multiple-year testing); numbers at the top of the plots represent the number of species included in that year. Lower panels: relationships within (*c*) and between (*d*) higher taxonomic groups are significant (*p* ≤ 0.03). Each filled circle represents one year. ‘Net population explosions’ represents the difference in numbers of species showing population explosions and crashes in a given year (e.g. if there are five species with an explosion and 15 with a crash in the same year, that year scores −10).

At least three extreme population changes took place in every year, revealing that every year in our four-decade study period was unusual from the perspective of some species (figure 2*a*,*b*). The majority of species experienced at least one extreme population change during their study periods: 86% of Lepidoptera (177 out of 207 species) and 93% of birds (29 of 31).

We detected a significant negative association between the proportion of Lepidoptera experiencing population crashes and the proportion experiencing population explosions across years (Spearman's rank correlation: *S* = 22 284.09, *r*_s_ = −0.57, *p* < 0.0001), indicating that when multiple species did exhibit extreme changes in the same year, they tended to respond in the same direction. This was not significant for birds (*S* = 13 689.1, *r*_s_ = −0.11, *p* = 0.49). Extreme population changes were, nonetheless, expressed in different directions in 41 of the 44 years considered (i.e. the populations of some species crashed and others exploded in the same year). Furthermore, even in the most extreme years (see below), most species did not exhibit extreme population responses, demonstrating the individualistic nature of the extreme population changes exhibited by species.

Out of a possible 10 178 species-by-year combinations, 374 (3.7%) population crashes and 257 (2.5%) population explosions were detected: an excess of crashes over explosions (two-tailed exact binomial test, *n* = 631, *p* < 0.001). Crashes also tended to be larger in their absolute magnitudes than explosions in both Lepidoptera (Welch two-sample *t*-test: *t* = −3.82, d.f. = 454.05, *p* < 0.001) and birds (*t* = −2.41, d.f. = 116.71, *p* < 0.02). For Lepidoptera, crashes (mean = −0.52, range −1.03 to −0.22) were on average around 13% greater in magnitude than explosions (mean = 0.46, range 0.21 to 1.30). Similarly for birds, crashes (mean = −0.13, range −0.48 to −0.03) were on average 18% greater in magnitude than explosions (mean = 0.11, range 0.04 to 0.23).

The numbers of extreme population changes in a given year for moths were strongly positively correlated with the numbers of extreme population changes in the same year for butterflies (Spearman's correlation: *S* = 3098.72, *r*_s_ = 0.60, *p* < 0.0002; figure 2*c*), suggesting that common external drivers were responsible for population crashes and explosions in Lepidoptera. However, comparing Lepidoptera and birds revealed a negative correlation (*S* = 16 433.1, *r*_s_ = −0.33, *p* = 0.03; figure 2*d*), suggesting that birds and Lepidoptera are responding to different external drivers, or to similar drivers but with different lagged responses.

The existence of common drivers that acted across multiple species was supported by the detection of five ‘consensus’ years for Lepidoptera (1975/1976, 1976/1977, 1992/1993, 2006/2007 and 2011/2012) during which statistically unusual numbers of species showed population explosions or crashes (at *p* < 0.05, after Bonferroni correction). Only one of these (1975/1976) was a consensus good year, while the other consensus years were generally bad years, during which nearly all extreme population changes (54 out of 59 in 1976/1977, 25 out of 26 in 1992/1993, 30 out of 32 in 2006/2007 and 42 out of 42 in 2011/2012) were negative (figure 2*a*). However, even during their largest consensus years, only 28% of Lepidoptera species and 32% of bird species experienced extreme population responses.

By contrast, for birds, only one consensus year was detected (1981/1982) as statistically significant (*p* < 0.05, after Bonferroni correction; 1990/1991 was significant prior to correction), during which 10 of the 31 species crashed and none exploded (figure 2*b*). The lower numbers of bird species compared with Lepidoptera in our analyses (31 rather than 207 species) may explain this apparent difference in number of consensus years between taxa, and so it should not be deduced that birds necessarily experienced fewer consensus years than Lepidoptera.

At a species-specific level, there were 38 cases across the study period (for seven birds, five butterflies and 21 moths) when an extreme population explosion was preceded by an extreme population crash, which represents 15% of the 257 population explosions that happened in total. Similarly, there were 31 cases (for two birds, five butterflies and 21 moths) when an extreme population crash was preceded by an extreme population explosion, representing 8% of the 374 population crashes. These may represent some combination of density-dependence, delayed climatic effects, delayed climatic effects mediated by density dependence, and coincidence when favourable conditions were followed by unfavourable conditions, or vice versa.

### (b) Associations between biological and climatic extremes

Five of the six consensus years for extreme population change coincided with one of the extreme climate years, either directly (*n* = 3) or with a 1-year lag, which is consistent with the hypothesis that there is a positive association between population consensus years and extreme climatic conditions (Fisher's Exact-Boschloo test, one-sided *p* = 0.015). The sixth consensus year for population change (1992/1993), which was the smallest of the consensus population crashes (figure 2), was not associated with any climatic extremes (table 1).

**Figure 3.**
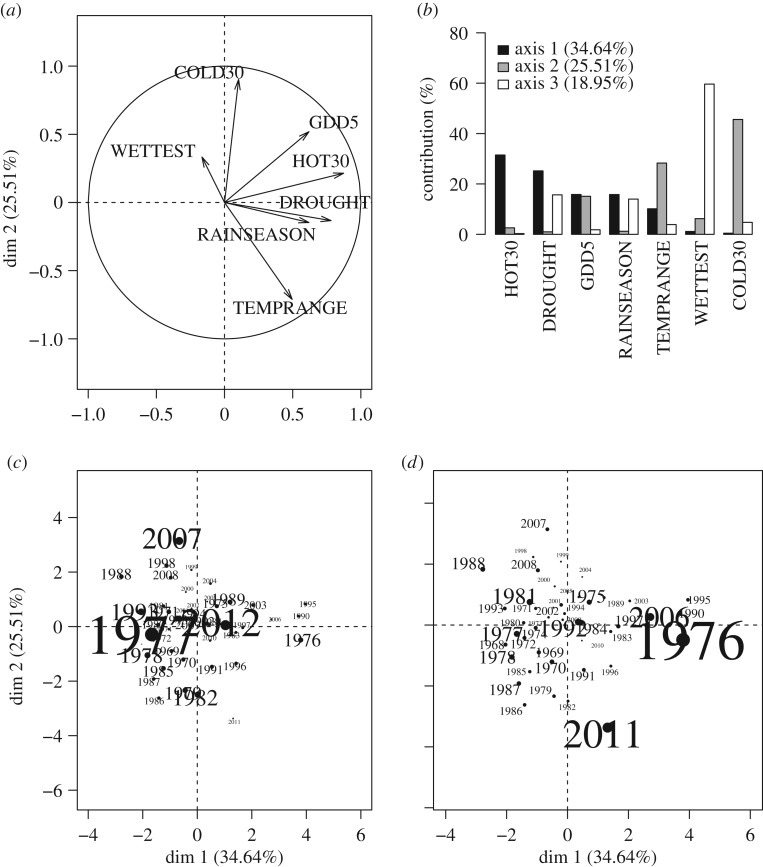
Principal components analysis (PCA) illustrating the variation in the seven climate variables (table 1) across our study period. (*a*) Vectors for individual climate variables associated with the first two PCA axes (i.e. dimensions, labelled ‘dim’); (*b*) the percentage contributions of each variable to the first three PCA axes. (*c*,*d*) The positions for each year on the first two axes; the size of the text reflects the relative size of the consensus year (i.e. the number of species experiencing an extreme population change) in either the year during which the population change was measured (*c*) or in the previous year (i.e. accounting for a 1-year population lag, (*d*)).

In the only consensus year for birds (1981/1982), 32% (10 of 31 species) of species crashed during exceptionally cold winter weather in that year (table 1 and figures 2 and 3). In 2006/2007, the large consensus year for Lepidoptera coincided with high growing degree days in that year, as well as an extremely hot summer in the previous year (i.e. 2005/2006; table 1 and figures 2 and 3). The large number of Lepidoptera crashing in the 2011/2012 consensus year followed extreme cold in the previous winter.

The one consensus good year for populations was 1975/1976, when 9% (*n* = 16) of moths experienced population explosions (butterflies could not be considered because data collection did not start until the following year) and none crashed. The climate in 1975 was relatively dry, with the summer of 1976 being extremely hot and dry (table 1 and figure 3*c*,*d*) with a drought index nearly double the median over the study period (figures 2*a*, 3*d* and table 1). Subsequently, significant numbers of Lepidoptera (54 of 207 species, 26%) experienced population crashes between 1976 and 1977. However, while 1976/1977 was the year with the most Lepidoptera crashes (54 of 207 species), a few Lepidoptera (four species) still experienced population explosions in the same year. This suggests that there can be cumulative effects, and that some climatic extremes may generate opposite direct and lagged effects (in this case, explosion followed by crash).

Five of the 10 climatically extreme years (1978/1979, 1985/1986, 1989/1990, 1994/1995 and 1995/1996) did not coincide, with or without lag, with any of the consensus population change years in either Lepidoptera or birds. Given that extreme events tended to happen in different years for Lepidoptera and birds (figure 2*d*), it is possible that other taxa responded strongly in these years. The pattern of apparently mixed responses is also exhibited by individual species. For example, the mottled grey moth *Colostygia multistrigaria* population crashed after the 1976 drought, but not after other dry years, and the tree sparrow *Passer montanus* declined in association with some, but not all, cold winters (figure 1).

**Figure 4.**
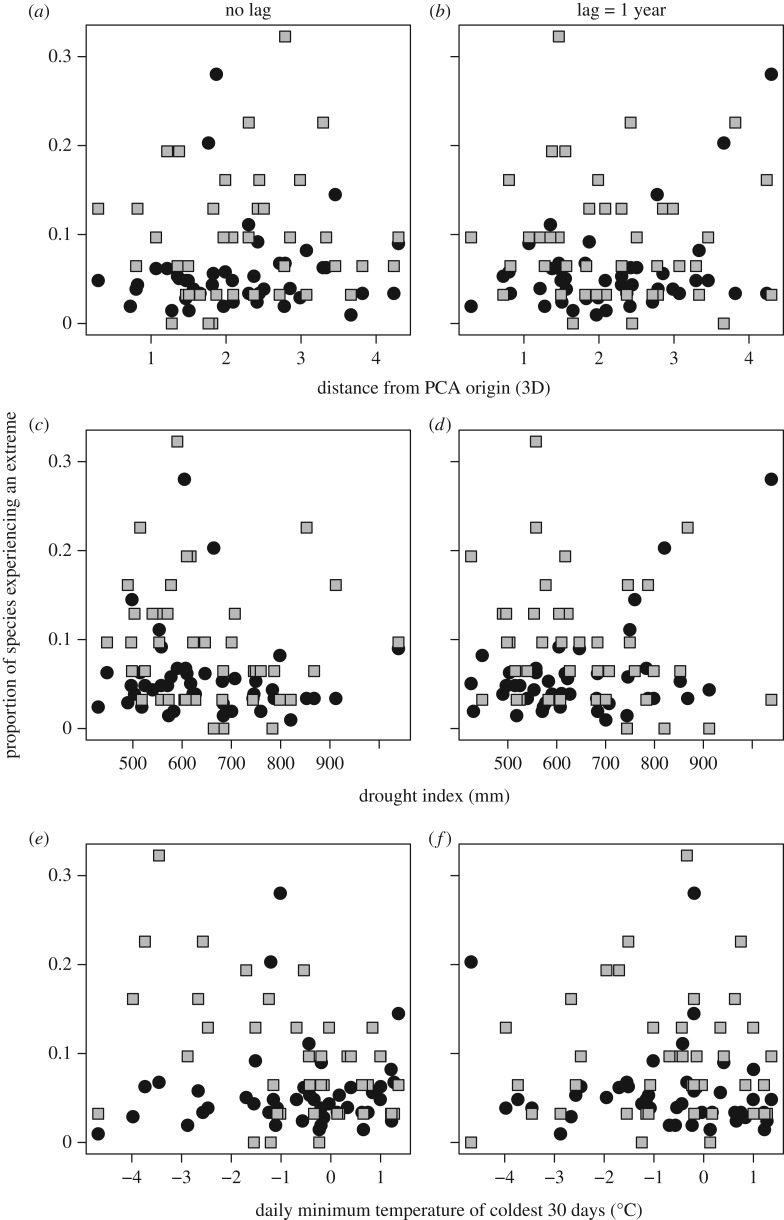
No overall relationship was observed between climatic conditions and the numbers of species showing extreme population responses. Relationships between the proportion of species experiencing an extreme response (either population crashes or explosion) in each year and three-dimensional distance from the climate-PCA origin (*a*,*b*), drought index (*c*,*d*) and daily minimum temperature of the coldest 30 days (*e*,*f*) are shown. Lepidoptera are represented by black circles and birds by grey squares; each symbol represents 1 year. The lags are measured in years, with lag 0 representing the climate measured in the current year, i.e. population changes from 1968–1969 were related to the climate in 1968 (lag = 1 year) and/or 1969 (no lag).

We then considered extreme population changes in all years in relation to PCA scores, drought and winter cold. There was no correlation between three-dimensional distance from the PCA origin (a measure of how climatically unusual a year was) and the proportion of species experiencing an extreme event (figure 4). The relationships between species' responses, drought and winter cold were also noisy for both Lepidoptera and birds (figure 4), with only two significant relationships detected after Bonferroni correction. The first significant relationship was for drought index of the previous year and the proportion of Lepidoptera species experiencing an extreme change (*t*_41_ = 3.30, *r* = 0.48, *p* = 0.002; figure 4*d*). The second was a significant negative correlation between the proportion of birds experiencing an extreme population change and daily minimum temperature of the coldest 30 days (*t*_39_ = −3.48, *r* = −0.49, *p* = 0.001; figure 4*e*). However, in both cases, the correlations ceased to be significant (after Bonferroni correction) once the largest consensus year was removed (1976/77 for Lepidoptera, *t*_40_ = 1.45, *r* = 0.22, *p* = 0.15; 1981/82 for birds, *t*_38_ = −2.81, *r* = −0.41, *p* = 0.01). This reinforces the view that consensus years are genuinely unusual. In the analyses above we reported the proportion of species experiencing an extreme change (both explosion and crash), but results were qualitatively the same when analysing those experiencing crashes or explosions, separately (see electronic supplementary material, figures S1 and S2, respectively).

### (c) Extremes and long-term population trends

**Figure 5.**
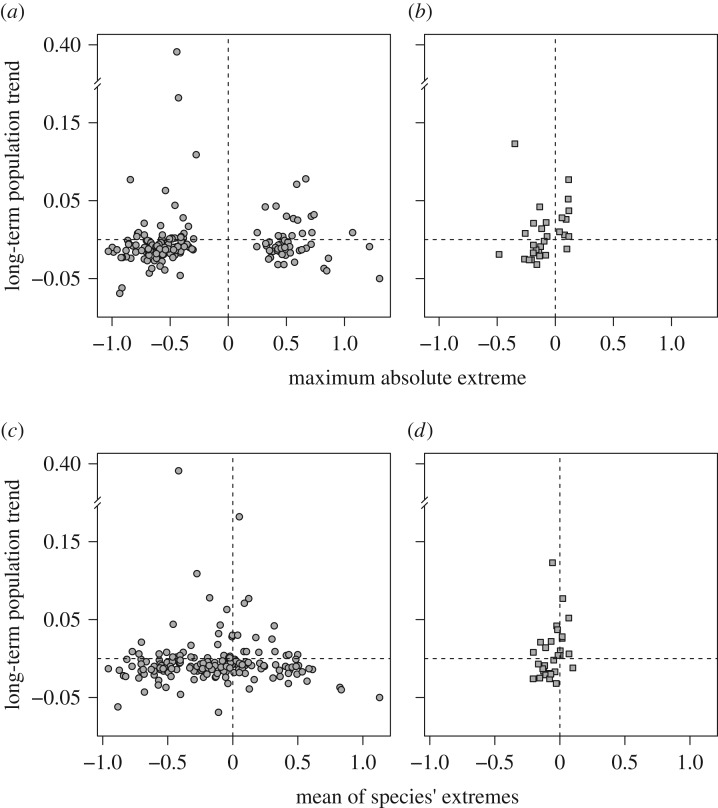
Relationships between Lepidoptera (*a*,*c*) and bird (*b*,*d*) species' long-term population trend and the maximum absolute extreme value for a species during the study period (*a*,*b*) and mean over all extreme events experienced by that species during the study period (*c*,*d*). Note the broken *y*-axes.

Overall, there was little relationship between the extreme population changes that a species exhibited and species' long-term population trends (figure 5). Extreme population events are modest predictors of long-term trends, at best, and for the Lepidoptera in our study may not be linked at all.

For Lepidoptera, we first compared two groups of species: those for which the single most extreme event was a crash, and those for which the single most extreme event was a population explosion. We found no association between extreme population change and trend (one-tailed Wilcoxon rank sum test: *W* = 3439.5, *p* = 0.19; figure 5*a*). We then took the mean of all extreme events exhibited by each species. Again, there was no difference between the long-term population trends of ‘crashing’ and ‘exploding’ species (*W* = 3583, *p* = 0.45; figure 5*c*). Regardless of the direction and magnitude of the extreme, some species showed long-term increases, and others showed long-term declines.

When we repeated this analysis for birds, we did find an effect of extreme events. We found that bird species experiencing population explosions (as single events, or the mean of their species-specific extremes) tended to have more positive long-term population trends than bird species that exhibited crashes (for single events, *W* = 144.5, *p* = 0.005 (significant after Bonferroni correction); average of all extremes, *W* = 128.5, *p* = 0.02 (n.s. after Bonferroni correction); figure 5). As in the Lepidoptera, some crashing bird species showed long-term population increases and others decreases. The different results for Lepidoptera and birds suggest that there may be taxonomic differences (perhaps linked to generation times) in the association between extreme events and long-term trends.

## 4. Discussion

### (a) The frequencies and magnitudes of extreme population events

Extreme population responses were observed in all years, and in at least 1 year for the majority of species: moths, butterflies and birds. Furthermore, in the majority of years, one or more species showed extreme positive population growth (explosions) while others simultaneously showed rapid declines (crashes). These findings show that extreme population changes are individualistic among species; an extreme year for one species is not necessarily an extreme year for another. Individualism can be expressed not only in the particular climate variables (or other drivers) that a species responds to, but also in the time delays between an event and the population response. The observed effects can be direct (e.g. population growth within a warm year), delayed by a year (e.g. drought-induced mortality of Lepidoptera that is not recorded until adults fail to emerge the next year), or delayed by 2 or more years via community interactions (e.g. through altered natural enemy or food abundances) [39]. Delayed density dependence (population crash following a good year, or vice versa) may add further lags to the system. Across all 238 species, a combination of delayed community and density-dependent effects could mean that extreme population responses are more evenly spread across years than the ECEs that may trigger these changes. The longer generation times, larger body size, higher trophic level (on average) and homeothermic biology of birds, compared with Lepidoptera, may tend to spread their observed responses more evenly across the years, as we observed. The (weak) negative correlation between the responses of birds and Lepidoptera (figure 2*d*) may stem from different lag times, differences in which aspects of environmental variation they respond to, and different overall sensitivities to the climate.

Although species generally differed in the years they found to be extreme, there was some agreement across species. First, there was evidence that species groups as a whole tended to respond in the same direction in a given year (i.e. experiencing either crashes or explosions), presumably in response to the same (climatic) drivers. Second, we detected six ‘consensus years’ in which a statistically significant excess of species exhibited crashes or population explosions. Furthermore, each of these years was characterized by near unanimity in the direction of the extreme population response. Although we should be cautious in interpreting five consensus (generally) bad years to one consensus good year as an excess of negative extreme events, we also found significantly more (by 46%) crashes than population explosions across the entire dataset. These observations are consistent with the hypothesis that more bad than good events are expected when the climate is changing rapidly. If populations show some degree of local adaptation to historical conditions, they may show extreme population collapses under novel conditions (even if they subsequently recover through adaptation to the new conditions).

There was also a tendency for the magnitudes of crashes to be greater than the magnitudes of increases. We interpret this as arising because it is, in principle, possible for all individuals within a large population to die simultaneously when they experience an extreme event, whereas population growth is constrained by the intrinsic rate of increase of a species. Nonetheless, for insects, the potential fecundity of individuals is high, and so extreme population growth can occasionally be achieved, especially for species that can accomplish multiple generations within a single year.

Overall, we conclude that a few species exhibit extreme population changes in most years, and that most species show extreme population changes in some years, but that there are some years that are characterized by excesses of dramatic population changes. Furthermore, there is an excess of population crashes, relative to explosions and there is a tendency for crashes to be larger in magnitude than increases.

### (b) The link to climate

Linking all of these extreme population changes to variation in the climate is difficult, given that extreme population responses took place in every year and lagged responses can occur. Moreover, some population explosions and crashes may have nothing to do with the climate, or with the interaction between the climate and other species. Biological interactions that take place within communities, including exaggerated (over-compensating) responses to density-dependent interactions, can potentially generate population fluctuations in the absence of external drivers.

However, there are several lines of evidence that lead us to suggest that the majority of the rapid changes observed here do stem from a geographically widespread external driver, with climate the most likely candidate. First, the year-to-year population crashes and explosions that we detected took place at a national scale (England). These are unlikely to be driven by more local factors, such as local habitat change, or local interactions between species that are unrelated to a widespread driver. Second, we found a strong positive correlation between the responses of our two groups of Lepidoptera (butterflies and moths) across years (figure 2*c*), and a negative correlation between Lepidoptera and birds (figure 2*d*). Given that the recording schemes for these three groups are independent, these correlations imply responses to climate events that are both geographically widespread and capable of generating between-year changes. Thirdly, the existence of statistically significant consensus years (and general agreement on whether these years are good or bad) again implies that some relatively fast-acting underlying causation is operating at the geographical scale of the whole of England. Changes in land-use and habitat management (which affect micro-climate), pesticides, the arrival of invasive species, and other drivers that contribute to longer-term trends are unlikely to act so broadly in a single year; it is only their interactions with widespread climatic factors that are likely to drive such effects [40]. We conclude that most (but not all) of the extreme population crashes and explosions that we have detected stem directly or indirectly from a near-synchronous, geographically widespread process, which is most likely to be the climate.

In general, we demonstrated an overall lack of association between climate and population responses across all years. However, we did find that consensus years (when many species showed extreme changes) were more likely to occur in years that were also extreme from a climatic perspective. With such rare events (six consensus years), we should be cautious about attributing them to specific climatic conditions. Nonetheless, five of the six consensus years appear to be associated with either cold winters (historic extremes that may be becoming less frequent and extreme), and with hot and dry summers (extremes that may increase in frequency and strength). Similarly, when we looked across all extreme responses rather than just the consensus years, we found associations with drought (for Lepidoptera) and winter cold (for birds). It should be noted that there were some years which were climatically extreme but did not generate biological consensus years; but given that birds and Lepidoptera differed in their dynamics (i.e. responding most strongly in different years) it is entirely feasible that other taxa that we did not study responded strongly in those years.

Three of the six biological consensus years took place in the same year as a climatic extreme, but the negative effects of hot and dry conditions in 1976, and of extreme winter cold in 2010/2011, were mainly observed as lagged population responses (around a quarter of the Lepidoptera species crashed in 1976/1977). The summer of 1976 was hot, and also experienced the greatest drought index in the 45-year time series, owing to hot and dry conditions stretching back to the spring/summer of 1975 (figure 1). This apparent lag in Lepidoptera response may be an issue of detection rather than a true biological phenomenon; individuals may have died in the summer of 1976, but it was not until the 1977 generation failed to emerge that this was noticed. For example, numbers of the Adonis blue butterfly *Polyommatus bellargus* crashed after its host plant *Hippocrepis comosa* dried up and caterpillars then starved [41]; and other species with summer-feeding larvae were also negatively affected [42]. The ringlet butterfly *Aphantopus hyperantus* also crashed [43] and so it seems likely that direct effects of the 1976 drought were largely responsible for the subsequent population crashes of other Lepidoptera. Impacts of summer drought conditions upon birds are likely to be weaker than for Lepidoptera (bird populations did not change abnormally in 1975/1976 or 1976/1977), although there is some previously documented evidence for lagged effects on some bird species that feed on soil invertebrates (e.g. [39]) as well as on those that are migrants [10].

### (c) Are population trends determined by extreme events?

It would seem reasonable to suppose that populations exhibiting major crashes would tend to decline in the long term, and those experiencing population explosions would increase. However, extreme events are rare, and many smaller population changes in ‘normal’ years might fully compensate for such extreme events. Density-dependent responses to extremes may also prevent any long-term consequences of extreme events from being realized. Our data suggest that any impact of single extreme events on long-term trends is limited (figure 5). In particular, for Lepidoptera and bird species experiencing population crashes (either as the most extreme event they experienced or as the average of all extreme events), some of them showed long-term declines and others showed long-term increases. The same was true for Lepidoptera that experienced population explosions. It was only in birds where species explosions tended to be linked to more positive long-term population trends.

There is no universal best way to test for the effects of extremes on long-term trends, but we urge others to test rather than assume that the two will be linked. Weak associations are not particularly surprising. Only 6.2% of all between-year population changes qualified as extreme, and hence the magnitude of extreme events would have to be far greater than regular population changes for such events to leave a strong signature on the overall population trend. Reducing the threshold for detecting extremes (so there are many more of them) might increase the likelihood of detecting an association, but this would be counter to the notion that extreme events are, by definition, unusual. Altwegg *et al*. [12] report that long-term observational studies of the impacts of extreme climatic events have tended to observe two or three extreme events during a median study duration of 10 years, which is comparable with the frequency of extreme population responses identified here. Of course, single events that reduce population densities by two or more orders of magnitude can happen [18,26], but they are very rare when considering the number of between-year population changes that we studied. Long-term population trends are seemingly dominated by other factors, such as relatively gradual climatic changes, or by non-climatic events that accumulate over space and time. For example, many farmland birds showed declining trends during the 1970s and 1980s as a result of agricultural intensification operating over many years [44,45]. Similarly, land-use change is the likely driver of the parallel long-term declines of many Lepidoptera species in the United Kingdom [46,47]. In no single year would there be sufficient intensification to cause a detectable crash at a national scale, but the accumulation of local effects over many years seems to drive the long-term trend. Other factors such as the arrival of invasive species or other locally acting pressures can have similar effects provided they operate for long enough; multiplicative effects of climatic and non-climatic factors may also be important [40].

An additional reason why a link between extreme population events and long-term trends may not be apparent could be related to historical extreme events (constraints) that are no longer in operation. Climate warming may be just as likely to reduce or remove some historical constraints as to impose new ones. For example, the insectivorous Dartford warbler *Sylvia undata* was virtually extinguished from England by the severe winter conditions of 1961/1962 [48], but this bird species has subsequently increased in abundance and expanded its distribution in the absence of such a severe winter cold constraint [14]. Dartford warblers still do worse in cold winters, but these temperatures are now insufficiently cold to determine the overall population trend. This phenomenon would lead to little or no correlation across species in their most extreme population responses and their overall population trends. Species may be released from historical constraints (including extremes), just as they may be hampered by novel ones.

## 5. Conclusion

In every year of our time series, at least three species of Lepidoptera and/or birds showed an extreme response in population size, and some species experienced extreme population crashes while others simultaneously experienced extreme population explosions in nearly every year. These findings give support to our first hypothesis—that the responses of species to climatic variability are individualistic (i.e. most years are associated with extreme population changes in some species). We also found support for our second hypothesis: population crashes tended to be more frequent than population explosions during periods of rapid climatic change (as new conditions are experienced by populations that are potentially locally adapted to historical conditions). Furthermore, population crashes were more extreme than explosions (explosions are constrained by the intrinsic rate of population growth whereas it is possible for all individuals to die). Thirdly, we did find that there were six statistically unusual consensus years when many species experienced extreme population changes, and we obtained support for the hypothesis that these events were associated with climatically extreme years. Finally, we found only limited and weak support (among birds) for the hypothesis that long-term population trends are correlated with extreme population responses, probably because the processes that are operating in most years (which are not extreme) are usually more important determinants of long-term trends than are rare extremes. We conclude that extreme population events are individualistic despite occasional consensus years, and are likely to be linked to climatic extremes (from the perspective of each species), but that these extreme events are only weak predictors of long-term population trends for the taxa we consider.

## Supplementary Material

Supplementary tables and figures

## Data accessibility

The raw data for these analyses are available from the organizations listed in the acknowledgements. Bird data are available via: www.bto.org/research-data-services/data-services/data-request-system, and butterfly data via: www.ukbms.org/Obtaining.aspx. Electronic supplementary material, table S4, contains the number of species, broken down by taxon, experiencing extreme population changes in each year.
