# Supplementary material for: Climate change, climatic variation and extreme biological responses
Source: Philos Trans R Soc Lond B Biol Sci. 2017 May 8;372(1723):20160144. doi: 10.1098/rstb.2016.0144 (PMC5434095; doi:10.1098/rstb.2016.0144)
Supplement: Supplementary tables and figures [file rstb20160144supp1.pdf]

Climate change, climatic variation, and extreme biological responses

Supplementary Table 1: Names of the 238 species included in the analysis.

Group	Latin name	Common name
Bird	*Aegithalos caudatus*	Long-tailed Tit
	Alauda arvensis	Skylark
	Buteo buteo	Buzzard
	Certhia familiaris	Treecreeper
	Chloris chloris	Greenfinch
	Columba palumbus	Woodpigeon
	Corvus corone	Carrion Crow
	Corvus monedula	Jackdaw
	Cyanistes caeruleus	Blue Tit
	Dendrocopos major	Great Spotted Woodpecker
	Emberiza calandra	Corn Bunting
	Emberiza citrinella	Yellowhammer
	Emberiza schoeniclus	Reed Bunting
	Erithacus rubecula	Robin
	Fringilla coelebs	Chaffinch
	Garrulus glandarius	Jay
	Parus major	Great Tit
	Passer montanus	Tree Sparrow
	Perdix perdix	Grey Partridge
	Periparus ater	Coal Tit
	Pica pica	Magpie
	Picus viridis	Green Woodpecker
	Poecile montana	Willow Tit
	Poecile palustris	Marsh Tit
	Prunella modularis	Dunnock
	Pyrrhula pyrrhula	Bullfinch
	Regulus regulus	Goldcrest
	Sitta europaea	Nuthatch
	Strix aluco	Tawny Owl
	Sturnus vulgaris	Starling
	Troglodytes troglodytes	Wren
Moth	*Abraxas grossulariata*	Magpie
	Abrostola tripartita /triplasia agg.	(Dark) Spectacle agg.
	Achlya flavicornis	Yellow-horned
	Agriopis aurantiaria	Scarce Umber
	Agriopis marginaria	Dotted Border
	Agrochola circellaris	Brick
	Agrochola litura	Brown-spot Pinion
	Agrochola lota	Red-line Quaker
	Agrochola lychnidis	Beaded Chestnut

© The Authors under the terms of the Creative Commons Attribution License http://creativecommons.org/licenses/by/3.0/, which permits unrestricted use, provided the original author and source are credited.

Group	Latin name	Common name
	Agrochola macilenta	Yellow-line Quaker
	Agrotis clavis	Heart & Club
	Agrotis exclamationis	Heart & Dart
	Agrotis puta	Shuttle-shaped Dart
	Alcis repandata	Mottled Beauty
	Allophyes oxyacanthae	Green-brindled Crescent
	Alsophila aescularia	March Moth
	Amphipyra tragopoginis	Mouse Moth
	Anticlea badiata	Shoulder Stripe
	Anticlea derivata	Streamer
	Apamea crenata	Clouded-bordered Brindle
	Apamea lithoxylaea	Light Arches
	Apamea monoglypha	Dark Arches
	Apamea remissa	Dusky Brocade
	Apamea sordens	Rustic Shoulder-knot
	Apeira syringaria	Lilac Beauty
	Arctia caja	Garden Tiger
	Asteroscopus sphinx	Sprawler
	Atethmia centrago	Centre-barred Sallow
	Autographa pulchrina	Beautiful Golden Y
	Axylia putris	Flame
	Biston betularia	Peppered Moth
	Biston strataria	Oak Beauty
	Brachylomia viminalis	Minor Shoulder-knot
	Cabera exanthemata	Common Wave
	Cabera pusaria	Common White Wave
	Calliteara pudibunda	Pale Tussock
	Campaea margaritata	Light Emerald
	Camptogramma bilineata	Yellow Shell
	Caradrina morpheus	Mottled Rustic
	Cerapteryx graminis	Antler Moth
	Cerastis rubricosa	Red Chestnut
	Charanyca trigrammica	Treble Lines
	Chloroclysta citrata	Dark Marbled Carpet
	Chloroclysta truncata	Common Marbled Carpet
	Chortodes pygmina	Small Wainscot
	Cidaria fulvata	Barred Yellow
	Cilix glaucata	Chinese Character
	Colocasia coryli	Nut-tree Tussock
	Colostygia multistrigaria	Mottled Grey
	Colostygia pectinataria	Green Carpet
	Colotois pennaria	Feathered Thorn
	Conistra ligula	Dark Chestnut
	Conistra vaccinii	Chestnut
	Cosmia trapezina	Dun-bar
	Cosmorhoe ocellata	Purple Bar
	Crocallis elinguaria	Scalloped Oak

Group	Latin name	Common name
	Cryphia domestica	Marbled Beauty
	Cybosia mesomella	Four-dotted Footman
	Diachrysia chrysitis	Burnished Brass
	Diaphora mendica	Muslin Moth
	Diarsia brunnea	Purple Clay
	Diarsia mendica	Ingrailed Clay
	Diarsia rubi	Small Square-spot
	Drepana falcataria	Pebble Hook-tip
	Ecliptopera silaceata	Small Phoenix
	Ectropis bistortata	Engrailed
	Eilema lurideola	Common Footman
	Electrophaes corylata	Broken-barred Carpet
	Ennomos alniaria	Canary-shouldered Thorn
	Ennomos quercinaria	August Thorn
	Epione repandaria	Bordered Beauty
	Epirrhoe alternata	Common Carpet
	Epirrita dilutata	November Moth
	Erannis defoliaria	Mottled Umber
	Eulithis pyraliata	Barred Straw
	Euplexia lucipara	Small Angle Shades
	Euproctis similis	Yellow-tail
	Eupsilia transversa	Satellite
	Euthrix potatoria	Drinker
	Falcaria lacertinaria	Scalloped Hook-tip
	Gortyna flavago	Frosted Orange
	Habrosyne pyritoides	Buff Arches
	Hemithea aestivaria	Common Emerald
	Hepialus humuli	Ghost Moth
	Hepialus lupulinus	Common Swift
	Hepialus sylvina	Orange Swift
	Herminia grisealis	Small Fan-foot
	Hoplodrina alsines	Uncertain
	Hoplodrina blanda	Rustic
	Hydraecia micacea	Rosy Rustic
	Hydriomena furcata	July Highflyer
	Hypena proboscidalis	Snout
	Idaea aversata	Riband Wave
	Idaea biselata	Small Fan-footed Wave
	Idaea dimidiata	Single-dotted Wave
	Idaea emarginata	Small Scallop
	Idaea seriata	Small Dusty Wave
	Idaea trigeminata	Treble Brown Spot
	Lacanobia oleracea	Bright-line Brown-eye
	Lacanobia thalassina	Pale-shouldered Brocade
	Laothoe populi	Poplar Hawk-moth
	Larentia clavaria	Mallow
	Laspeyria flexula	Beautiful Hook-tip

Group	Latin name	Common name
	Ligdia adustata	Scorched Carpet
	Lomaspilis marginata	Clouded Border
	Lomographa temerata	Clouded Silver
	Luperina testacea	Flounced Rustic
	Lycia hirtaria	Brindled Beauty
	Lycophotia porphyrea	True Lover's Knot
	Mamestra brassicae	Cabbage Moth
	Melanchra pisi	Broom Moth
	Mesapamea secalis/didyma	Common Rustic agg.
	Mesoligia furuncula	Cloaked Minor
	Mythimna comma	Shoulder-striped Wainscot
	Mythimna conigera	Brown-line Bright-eye
	Mythimna ferrago	Clay
	Mythimna impura	Smoky Wainscot
	Mythimna pallens	Common Wainscot
	Noctua comes	Lesser Yellow Underwing
	Noctua janthe	Lesser Broad-bordered Yellow Underwing
	Nola cucullatella	Short-cloaked Moth
	Notodonta ziczac	Pebble Prominent
	Ochropleura plecta	Flame Shoulder
	Odontopera bidentata	Scalloped Hazel
	Oligia fasciuncula	Middle-barred Minor
	Omphaloscelis lunosa	Lunar Underwing
	Operophtera brumata	Winter Moth
	Opisthograptis luteolata	Brimstone Moth
	Orthosia cerasi	Common Quaker
	Orthosia cruda	Small Quaker
	Orthosia gothica	Hebrew Character
	Orthosia gracilis	Powdered Quaker
	Orthosia incerta	Clouded Drab
	Orthosia munda	Twin-spotted Quaker
	Ourapteryx sambucaria	Swallow-tail Moth
	Peribatodes rhomboidaria	Willow Beauty
	Perizoma affinitata	The Rivulet
	Perizoma alchemillata	Small Rivulet
	Perizoma didymata	Twin-spot Carpet
	Perizoma flavofasciata	Sandy Carpet
	Petrophora chlorosata	Brown Silver-line
	Phalera bucephala	Buff-tip
	Pheosia gnoma	Lesser Swallow Prominent
	Phigalia pilosaria	Pale Brindled Beauty
	Photedes minima	Small Dotted Buff
	Phragmatobia fuliginosa	Ruby Tiger
	Plagodis dolabraria	Scorched Wing
	Poecilocampa populi	December Moth
	Pterostoma palpina	Pale Prominent
	Ptilodon capucina	Coxcomb Prominent

Group	Latin name	Common name
	Rusina ferruginea	Brown Rustic
	Scopula imitaria	Small Blood-vein
	Scotopteryx chenopodiata	Shaded Broad-bar
	Selenia dentaria	Early Thorn
	Selenia tetralunaria	Purple Thorn
	Spilosoma lubricipeda	White Ermine
	Spilosoma luteum	Buff Ermine
	Thalpophila matura	Straw Underwing
	Thera obeliscata	Grey Pine Carpet
	Tholera decimalis	Feathered Gothic
	Thyatira batis	Peach Blossom
	Timandra comae	Blood-vein
	Trichiura crataegi	Pale Eggar
	Tyria jacobaeae	Cinnabar
	Xanthia aurago	Barred Sallow
	Xanthia icteritia	Sallow
	Xanthia togata	Pink-barred Sallow
	Xanthorhoe designata	Flame Carpet
	Xanthorhoe ferrugata	Dark-barred Twin-spot Carpet
	Xanthorhoe fluctuata	Garden Carpet
	Xanthorhoe montanata	Silver-ground Carpet
	Xanthorhoe spadicearia	Red Twin-spot Carpet
	Xestia baja	Dotted Clay
	Xestia sexstrigata	Six-striped Rustic
	Xestia triangulum	Double Square-spot
	Xestia xanthographa	Square-spot Rustic
	Xylocampa areola	Early Grey
	Zanclognatha tarsipennalis	Fan-foot
Butterfly	*Aglais urticae*	Small Tortoiseshell
	Anthocharis cardamines	Orange-tip
	Aphantopus hyperantus	Ringlet
	Argynnis aglaja	Dark Green Fritillary
	Aricia agestis	Brown Argus
	Callophrys rubi	Green Hairstreak
	Celastrina argiolus	Holly Blue
	Coenonympha pamphilus	Small Heath
	Erynnis tages	Dingy Skipper
	Gonepteryx rhamni	Brimstone
	Hipparchia semele	Grayling
	Aglais io	Peacock
	Lasiommata megera	Wall
	Limenitis camilla	White Admiral
	Lycaena phlaeas	Small Copper
	Maniola jurtina	Meadow Brown
	Melanargia galathea	Marbled White
	Favonius quercus	Purple Hairstreak
	Ochlodes sylvanus	Large Skipper

Group	Latin name	Common name
	Pararge aegeria	Speckled Wood
	Pieris brassicae	Large White
	Pieris napi	Green-veined White
	Pieris rapae	Small White
	Polygonia c-album	Comma
	Polyommatus coridon	Chalk Hill Blue
	Polyommatus icarus	Common Blue
	Pyrgus malvae	Grizzled Skipper
	Pyronia tithonus	Gatekeeper
	Thymelicus sylvestris	Small Skipper

Supplementary Table 2: Full list of potential climate variables. Underlined abbreviations indicate the seven weakly-correlated (pairwise Pearson's $|r| < 0.7$) variables used in the analysis. With the exception of the drought index, each variable was calculated over the 12-month period from September 1 to August 31, to align with the end of the annual collection period of the species records. For the drought index, calculations ran over an 18-month period (beginning March 1) in order to capture water deficit accumulated over successive hot and dry springs/summers.

Variable	Abbreviation	Units	Description
Annual rainfall	RAINAN	mm/year	Annual sum of monthly rainfall values
Rainfall wettest month	WETTEST	mm/month	Rainfall of the wettest calendar month
Rainfall seasonality	RAINSEASON	mm	Rainfall contrast across seasons (13): $\sum s = 1..4\ \lvert R_s - R_T/4 \rvert / R_T$, where R_s is rainfall in season s, and R_T is total annual rainfall
Annual moisture index	MI	-	= RAINAN / Potential evapotranspiration
Drought index	DROUGHT	mm	Accumulated water deficit, where a deficit is defined by monthly Hargreaves PET > monthly rainfall. Months with excess rainfall reduce the deficit, but only up to field capacity. Drought index given by maximum water deficit recorded during spring/summer of reference year
Spring/summer sunshine	SUNSPRSUM	hours/day	Mean over March through August only
Growing degree days	GDD5	°C	Day-by-day sum of degrees by which daily mean air temperature exceeds 5 °C
Minimum temperature	MINTMP	°C	Minimum temperature across all days of the year
Maximum temperature	MAXTMP	°C	Maximum temperature across all days of the year
Annual temperature range	TEMPRANGE	°C	= MAXTMP – MINTMP
Isothermality	ISO	°C	= Diurnal temperature range / AN_TEMPRANGE
Daily minimum temperature of coldest 30 days	COLD30	°C	Mean of daily minima over coldest 30-day period
Daily maximum temperature of hottest 30 days	HOT30	°C	Mean of daily maxima over hottest 30-day period

Supplementary Table 3: Extreme years along the first three axes of the principal components analysis (see also Figure 3). Outliers are years in which the PCA coordinates are greater than ('positive extreme') or less than ('negative extreme') twice the median absolute deviation from the median.

PCA axis	Positive extreme	Negative extreme
1	1975/76, 1989/90, 1994/95, 2005/06	
2	1997/98, 2006/07	1978/79, 1981/82, 1985/86, 2010/11
3	1978/79, 2000/01	1971/2, 1975/76, 1988/89

Supplementary Table 4: The number of species, broken down by taxon (Butterfly, Moth, Bird), experiencing negative or positive extreme population changes in each year. The number of species analysed in each taxonomic group in each year is provided, along with the years identified as consensus years ('Y') for birds or Lepidoptera.

Year	Negative Extreme (#Butterflies)	Positive Extreme (#Butterflies)	Negative Extreme (#Moths)	Positive Extreme (#Moths)	Negative Extreme (#Birds)	Positive Extreme (#Birds)	Number of butterflies	Number of moths	Number of birds	Consensus year for birds	Consensus year for Lepidoptera
1968/69	NA	NA	5	6	1	2	0	178	31		
1969/70	NA	NA	5	4	5	1	0	178	31		
1970/71	NA	NA	9	2	1	5	0	178	31		
1971/72	NA	NA	5	2	2	1	0	178	31		
1972/73	NA	NA	1	9	2	2	0	178	31		
1973/74	NA	NA	5	0	0	2	0	178	31		
1974/75	NA	NA	5	6	1	2	0	178	31		
1975/76	NA	NA	0	16	2	1	0	178	31		Y
1976/77	12	0	42	4	0	1	29	178	31		Y
1977/78	1	7	3	8	3	1	29	178	31		
1978/79	2	2	1	8	6	1	29	178	31		
1979/80	2	0	7	1	0	2	29	178	31		
1980/81	3	1	4	2	0	1	29	178	31		
1981/82	1	7	0	6	10	0	29	178	31	Y	
1982/83	0	1	4	3	0	1	29	178	31		
1983/84	0	1	4	3	1	1	29	178	31		
1984/85	5	0	6	1	3	2	29	178	31		
1985/86	0	1	2	3	5	0	29	178	31		
1986/87	1	0	1	6	2	2	29	178	31		
1987/88	0	2	11	0	2	1	29	178	31		
1988/89	1	3	1	12	1	0	29	178	31		
1989/90	0	0	5	2	2	0	29	178	31		
1990/91	0	1	0	6	7	0	29	178	31		
1991/92	0	3	0	8	1	1	29	178	31		
1992/93	6	0	17	0	2	1	29	178	31		Y
1993/94	0	1	4	4	3	1	29	178	31		
1994/95	0	1	0	6	2	0	29	178	31		
1995/96	0	1	2	4	3	2	29	178	31		
1996/97	0	1	7	1	0	0	29	178	31		
1997/98	0	0	12	2	1	0	29	178	31		
1998/99	0	0	3	2	0	1	29	178	31		
1999/00	0	0	1	2	0	1	29	178	31		
2000/01	0	0	2	3	NA	NA	29	178	0		

© The Authors under the terms of the Creative Commons Attribution License http://creativecommons.org/licenses/by/3.0/, which permits unrestricted use, provided the original author and source are credited.

Year	Negative Extreme (#Butterflies)	Positive Extreme (#Butterflies)	Negative Extreme (#Moths)	Positive Extreme (#Moths)	Negative Extreme (#Birds)	Positive Extreme (#Birds)	Number of butterflies	Number of moths	Number of birds	Consensus year for birds	Consensus year for Lepidoptera
2001/02	0	0	3	1	NA	NA	29	178	0		
2002/03	0	1	0	10	1	0	29	178	31		
2003/04	0	0	2	5	0	1	29	178	31		
2004/05	0	0	3	0	0	0	29	178	31		
2005/06	0	0	1	3	2	0	29	178	31		
2006/07	3	0	25	2	2	0	29	178	31		Y
2007/08	0	0	4	6	2	1	29	178	31		
2008/09	0	1	1	8	3	1	29	178	31		
2009/10	0	0	1	3	0	3	29	178	31		
2010/11	0	0	2	0	1	0	29	178	31		
2011/12	5	0	37	0	0	0	29	178	31		Y

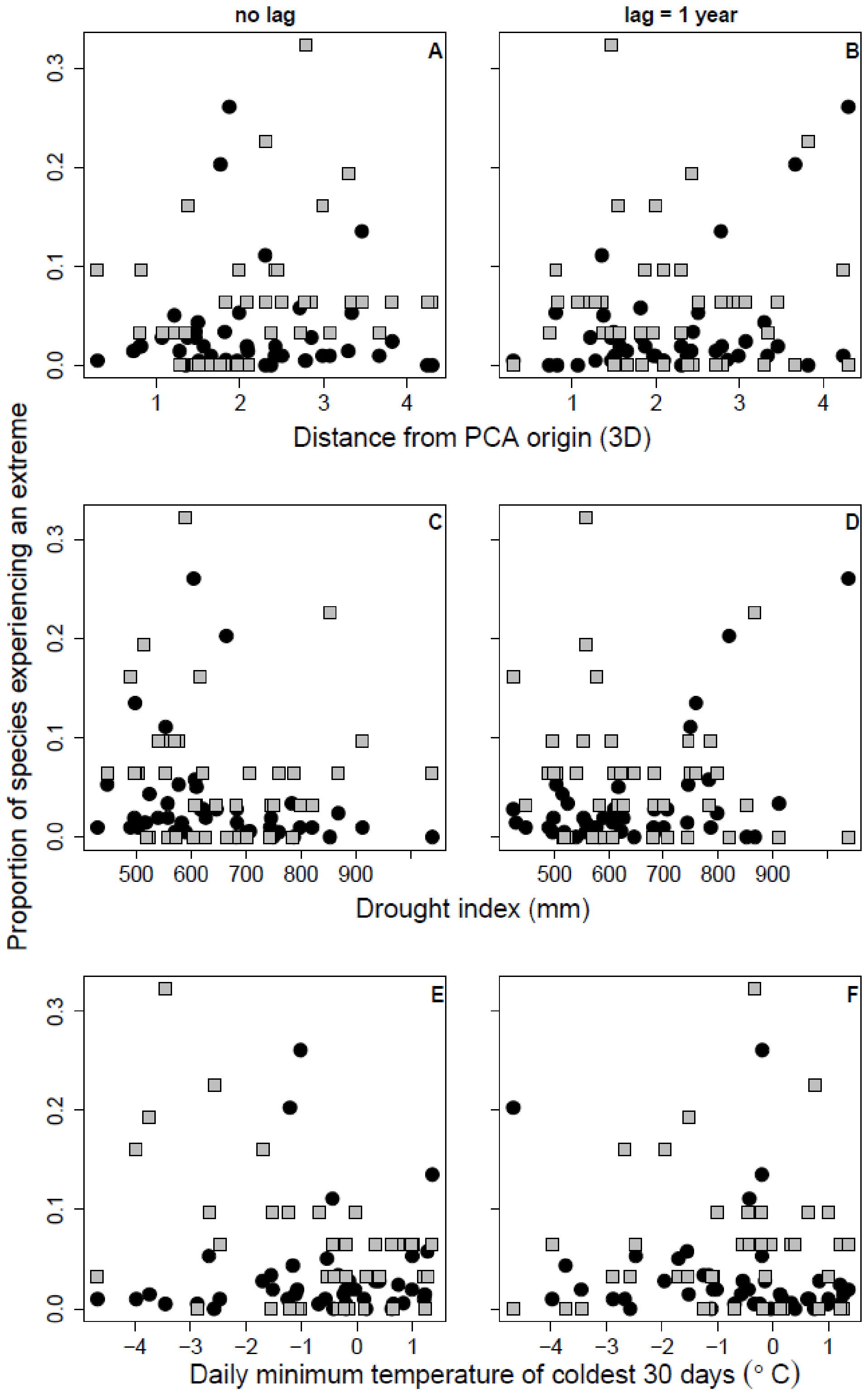

Supplementary Figure 1: Relationships between climate and species' population crashes. No overall relationship was observed between climatic conditions and the numbers of species showing extreme population crashes. Relationships between the proportion of species experiencing an extreme population crashes in each year and 3D distance from the climate-PCA origin (A, B), drought index (C, D) and daily minimum temperature of the coldest 30 days (E, F) are shown. Lepidoptera are represented by black circles and birds by grey squares; each symbol represents one year. The lags are measured in years, with lag 0 representing the climate measured in the current year i.e. population changes from 1968 – 1969 were related to the climate in 1968 (lag = 1 year) and/or 1969 (no lag).

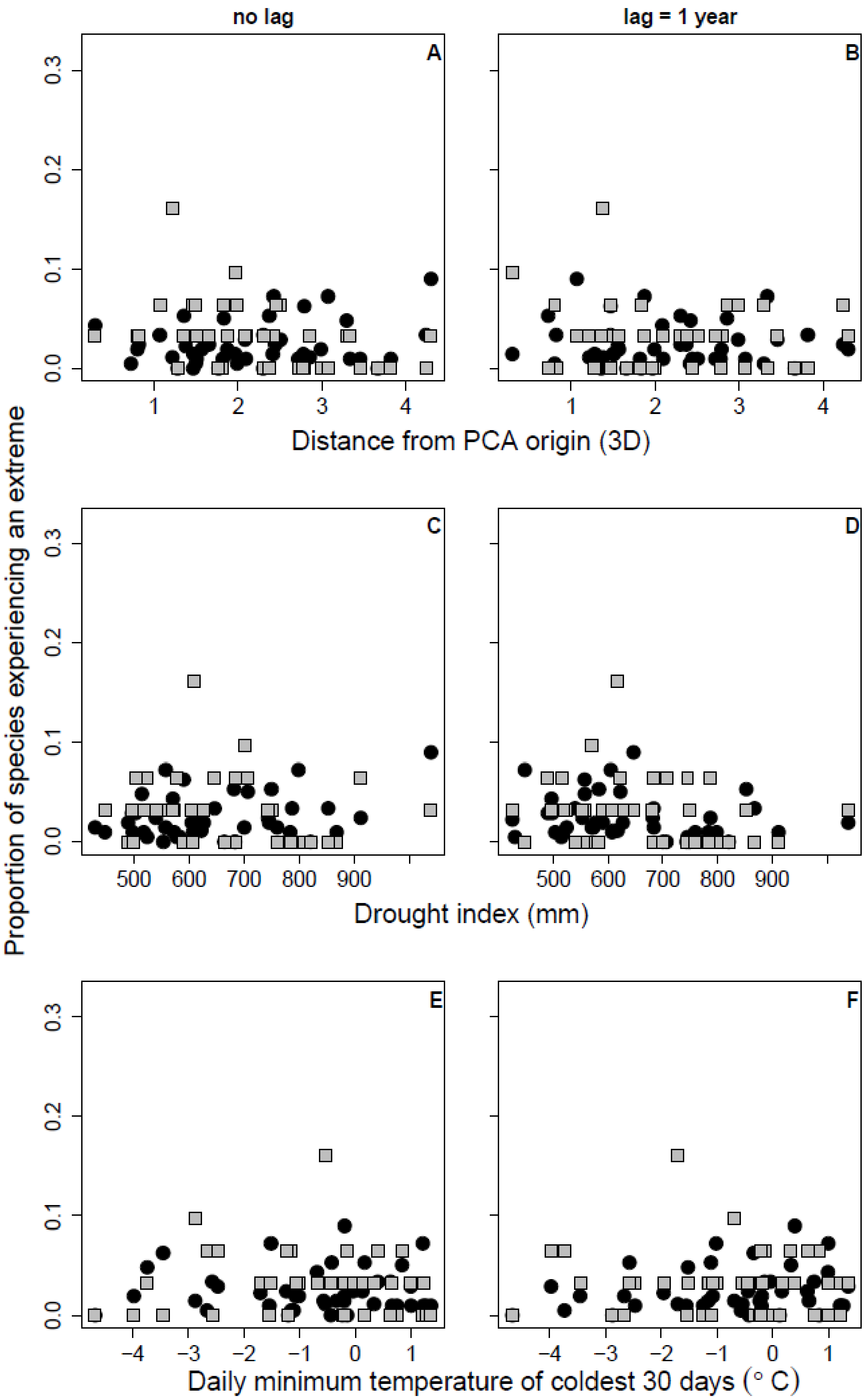

Supplementary Figure 2: Relationships between climate and species' population explosions. No overall relationship was observed between climatic conditions and the numbers of species showing extreme population explosions. Relationships between the proportion of species experiencing an extreme population explosions in each year and 3D distance from the climate-PCA origin (A, B), drought index (C, D) and daily minimum temperature of the coldest 30 days (E, F) are shown. Lepidoptera are represented by black circles and birds by grey squares; each symbol represents one year. The lags are measured in years, with lag 0 representing the climate measured in the current year i.e. population changes from 1968 – 1969 were related to the climate in 1968 (lag = 1 year) and/or 1969 (no lag).
